# Proteome and Nutritional Shifts Observed in Hordein Double-Mutant Barley Lines

**DOI:** 10.3389/fpls.2021.718504

**Published:** 2021-09-09

**Authors:** Utpal Bose, Angéla Juhász, Ronald Yu, Mahya Bahmani, Keren Byrne, Malcolm Blundell, James A. Broadbent, Crispin A. Howitt, Michelle L. Colgrave

**Affiliations:** ^1^CSIRO Agriculture and Food, St Lucia, QLD, Australia; ^2^Australian Research Council Centre of Excellence for Innovations in Peptide and Protein Science, School of Science, Edith Cowan University, Joondalup, WA, Australia; ^3^CSIRO Agriculture and Food, Canberra, ACT, Australia

**Keywords:** barley, *Hordeum vulgare*, *lys3* mutant, omics, hordein, coeliac disease, proteomics, SWATH-MS

## Abstract

Lysine is the most limiting essential amino acid in cereals, and efforts have been made over the decades to improve the nutritional quality of these grains by limiting storage protein accumulation and increasing lysine content, while maintaining desired agronomic traits. The single *lys3* mutation in barley has been shown to significantly increase lysine content but also reduces grain size. Herein, the regulatory effect of the *lys3* mutation that controls storage protein accumulation as well as a plethora of critically important processes in cereal seeds was investigated in double mutant barley lines. This was enabled through the generation of three hordein double-mutants by inter-crossing three single hordein mutants, that had all been backcrossed three times to the malting barley cultivar Sloop. Proteome abundance measurements were integrated with their phenotype measurements; proteins were mapped to chromosomal locations and to their corresponding functional classes. These models enabled the prediction of previously unknown points of crosstalk that connect the impact of *lys3* mutations to other signalling pathways. In combination, these results provide an improved understanding of how the mutation at the *lys3* locus remodels cellular functions and impact phenotype that can be used in selective breeding to generate favourable agronomic traits.

## Introduction

Barley is an important cereal grown mainly for feed and malting. The major seed storage proteins in barley, the hordeins, are elicitors of coeliac disease (CD) – a condition that adversely affects ∼1% of the world population (∼70 million people). There is no current treatment other than strict adherence to a life-long gluten-free (GF) diet. In the diploid barley genome, there are four types of hordeins present: B-, C-, D- and γ-hordeins. The B- and C-hordeins are the primary classes representing >90% of the hordeins in barley (Kreis et al., 1983; Shewry et al., 1985). The B- and C-hordeins are encoded by the *Hor-2* and *Hor-1* loci, respectively, and both loci are located on the short arm of chromosome 1. The high molecular weight glutenin orthologous D-hordein genes are located at *Hor-3* (1H long arm) and the S-rich γ-hordeins present at *Hor-5* (1H short arm) (Shewry, 1993). The B- and C-hordeins comprise two multigene families consisting of 13 B-hordein genes (Kreis et al., 1983) and 20–30 C-hordein genes, respectively (Shewry et al., 1985). Attempts have been made to reduce the hordein abundance in barley through mutagenesis, gene technology (antisense or RNAi), or by applying selective breeding techniques. The potential pleiotropic consequences of these mutations on the grain proteome are not well understood.

The high concentration of lysine-poor prolamin storage proteins in cereals are associated with the sub-optimal nutritional quality of cereal grains. Attempts have been made over the decades to increase the lysine content in cereal seeds by using chemical and physical mutagenesis. Early success was reported for two high lysine maize cultivars opaque-2 (Mertz et al., 1964) and floury-2 (Nelson et al., 1965). These two mutants had increased lysine and tryptophan content in the grain resulting from suppression of the lysine-deficient zein fraction without altering the contribution of other protein fractions. Screening efforts to identify similar *lys* mutants in other cereal grains led to the identification of a high-lysine gene in barley cultivars Hiproly (Munck et al., 1970) and Risø (Doll, 1973). Within the series of Risø mutants, Risø 1508 contained 45% more lysine and was shown to improve pig (Batterham, 1992) and rat (Gabert et al., 1995) growth in feeding trials without protein or amino acid supplementation. Although there was a significant down-regulation of hordeins (lysine-poor proteins) and an increase of free lysine, an increase in embryo size (as a proportion of grain size) compared to wild-types was significant (Tallberg, 1981); almost all Risø *lys* mutants had low starch content and shrunken grain size. The shrunken grain size was shown to relate to a set of genes involved in starch biosynthesis pathways (Cook et al., 2018; Moehs et al., 2019), but there is a lack of understanding of how the corresponding genes in the endosperm control starch content and composition. The abnormally large embryos in *lys3* mutants contain increased starch levels, increased starch granule size in the scutellum (Deggerdal et al., 1986) and have reduced dormancy (Howard et al., 2012). Although these mutants offer promise in terms of nutritional quality improvement, the concomitant reduction in seed size leading to lower agronomic yield negatively impacted their suitability for use as commercial crops.

Conventional breeding strategies were used to combine three recessive alleles to further reduce the hordein content in barley lines (Tanner et al., 2015). The high-lysine mutant Risø 56 barley line consists of a large gamma-ray-radiation-induced genomic deletion of at least 85 kb chromosome segment that accommodates the entire B-hordein loci (Kreis et al., 1983) on the short arm of chromosome 1H and does not accumulate the majority of the B-hordeins (Shewry et al., 1979). Another cultivar Risø 1508 contains an ethyl methanesulfonate (EMS)-induced mutation in the Lys3 locus on chromosome 5H (Karlsson, 1977). Risø 1508 has near zero C-hordein levels and significantly decreased B-hordein levels (Doll, 1973; Deggerdal et al., 1986; Hansen et al., 2007), confirmed by the transcription of the B- and C-hordein genes which are significantly down-regulated (Sørensen, 1992). An Ethiopian-derived landrace, R118 contains a single spontaneous mutation that encodes a premature stop codon that prevents expression of the full-length D-hordein (Tanner et al., 2015). Although several approaches have been used over the past decades to reduce hordein abundance in cereal crops, including antisense or RNAi, or selective breeding approaches (Hansen et al., 2007; Lange et al., 2007; Tanner et al., 2015; Moehs et al., 2019), the potential pleiotropic effects on the proteome upon mutation are not well understood.

The mechanisms activated in the grains of these double-mutant lines to compensate for the loss of these major storage proteins and decrease in starch content remain unknown. In the current study, phenotypic characterisation was performed in parallel with data-independent acquisition (DIA) mass spectrometry analyses (Venable et al., 2004), specifically sequential window acquisition of all theoretical spectra (SWATH)-mass spectrometry (Gillet et al., 2012). The aim was to study the large-scale quantitative changes in grain proteins within the hordein DM lines in comparison to their parent lines. Functional annotation and bioinformatic analyses were carried out to uncover the protein classes related to the hordein reduction in the DM lines. Balanced changes in the induced and suppressed protein abundances indicate differences both in the hordein levels and composition as well as in the lysine content of the DMs. This study serves as a framework for future proteomics-assisted crop development to study the pleotropic effects of genetic modification on safety and nutrition quality-related improvements.

## Materials and Methods

### Plant Materials

The hordein null lines used in this study were developed in a previous study (Colgrave et al., 2016). Briefly, the barley varieties Risø 56 (B-hordein null), Risø 1508 (C-hordein null), and Ethiopian R118 (D-hordein null) were each crossed with the standard malting grain cv Sloop four times followed by selfing generations to create hordein single-nulls at backcross 3 (BC3), which theoretically share 93.75% genetic identity with Sloop. One homozygous hordein single-null for each of the three loci was selected and those three lines were intercrossed to create all three possible combinations of the Sloop hordein double-nulls.

The lines have also been assessed by MS to confirm the absence of the hordeins confirming that these mutations have not arisen spontaneously during the crossing programme. A limitation of this experiment is that only one line of each SM was selected for further study, and mutant segregants from each inter-cross of the SM were not examined. Pools of seeds from SM and DM lines were milled separately for obtaining the flour samples for analysis. Milling the individual seed was avoided as this was expected to introduce losses from already limited sample during the milling process.

### Embryo Size Measurement

The size of the whole barley caryopsis was observed under stereomicroscopy (MZFIII, Leica Microsystems). The image was then processed by the image processing package Fiji in ImageJ (Supplementary File 1) (Schindelin et al., 2012).

### Barley β-Glucan Analysis

Barley β-glucan was measured according to AOAC Method 995.16 (McCleary and Draga, 2016). Samples representing 20 mg of barley flour underwent sequential enzymatic digestion with lichenase and β-glucosidase and the glucose released was quantified through the standard glucose oxidase/peroxidase (GOPOD) system (Supplementary File 1).

### Total Starch Content Analysis

The total starch content was measured using the AOAC Method 996.11 (AOAC, 1995), according to McCleary et al. (1997). The commercial K-TSTA kit (Megazyme) was used under manufacturer’s procedures (McCleary et al., 1997). Briefly, starch was hydrolysed by thermostable α-amylase and amyloglucosidase to D-glucose. Glucose was then determined and quantified with glucose oxidase-peroxidise reagent (Supplementary File 1).

### Total Fatty Acid and Total Triacyl Glycerol (TAG) Content Analysis

The extraction of total lipid, fractionation of neutral lipid (mainly TAG), free fatty acid, and polar lipid (mainly phosphocholine), and the subsequent lipid quantification were conducted according to the methods described by Liu et al. (2017). The quantitation of total lipid content and fatty acid composition were conducted based on the methods described by Vanhercke and co-authors in 2014 (Vanhercke et al., 2014). Detailed protocols have been described in Supplementary File 1.

### Protein Extraction and Digestion

Proteins were extracted from three biological replicates of wholemeal flour (100 mg) in 1 mL of 8 M urea, 2% (w/v) dithiothreitol (DTT). The solution was thoroughly vortexed and sonicated for 5 min until completely mixed. Protein reduction continued on a thermomixer block, shaking at 1,000 rpm at 22°C for 45 min. The solutions were centrifuged for 15 min at 20,800 × *g* and the supernatants were used for subsequent analysis. Protein estimations were performed using the Bio-Rad microtiter Bradford protein assay (California, United States) following the manufacturer’s protocol. The protein extracts were diluted in water over two dilutions (1:20, 1:40) in duplicate and measurements were made at 595 nm using a SpectraMax Plus (Molecular Devices). Bovine serum albumin (BSA) standard was used in the linear range from 0.05 to 0.5 mg/mL. The BSA standard concentration was determined by high sensitivity amino acid analysis at the Australian Proteomics Analysis Facility (Sydney, Australia). Blank-corrected standard curves were run in duplicate. Linear regression was used to fit the standard curve. Protein sample wash and digestion steps were performed as precisely described in Colgrave et al. (2016).

### LC-MS/MS Data Acquisition

Protein extracts were reconstituted in 100 μL of aqueous 0.1% formic acid/10.0% acetonitrile. The peptide fractions (5.0 μL) with iRT peptides (0.5 μL; Biognosys, Zurich, Switzerland) were chromatographically separated with an Ekspert nanoLC425 (Eksigent, Dublin, CA, United States) directly coupled to a TripleTOF 6600 MS (SCIEX, Redwood City, CA, United States). The peptides were desalted on an YMC Triart C18 (12 nm, 5.0 mm × 0.5 mm) trap column at a flow rate of 15 μL/min in solvent A and separated on an YMC Triart C18 (3 μm, 120 Å, 5.0 × 0.5 mm) column at a flow rate of 5 μL/min. The injection volume was 5.5 μL for the information-dependant acquisition (IDA). The analysis method and LC-MS/MS parameters were precisely described in Colgrave et al. (2016).

### SWATH-MS Data Acquisition and Library Generation

The digested protein extracts (2.5 μL; 2.0 μL of sample plus 0.5 μL of iRT peptide standard, Biognosys) were chromatographically separated as described for IDA. The MS source conditions were also identical. The TOF-MS survey scan was collected over the mass range of *m/z* 360-2000 with a 150 ms accumulation time and the product ion mass spectra were acquired over the mass range *m/z* 110-2000 with 30 ms accumulation time. Variable window SWATH ranges were determined using the SWATH variable window calculator 1.0 (SCIEX) to identify 30 optimal ranges (including 1 Da overlap) spanning *m/z* 360-2000 and resulting in a 1.1 s cycle time. Collision energy (CE) was determined using each window centre as the input *m/z* for CE equations and a CE spread of 5 eV used to allow for *m/z* variance across each SWATH window.

### SWATH-MS Data Processing for Peptide Quantitation

Targeted data extraction of SWATH files was performed using the MS/MS^ALL^ with SWATH Acquisition MicroApp v2.0 plug-in for PeakView v2.2 software (SCIEX). Retention time alignment was achieved using the iRT peptides. The peptide ion library was generated by searching IDA data against the Poaceae subset of the UniProt-KB database (2017/05; appended with a custom gluten database and the iRT peptides; 1,437,912 sequences). Protein and peptide identifications were filtered at a 1% false discovery rate (FDR) during input of the search result to PeakView. The processing settings were a maximum of 100 peptides per protein with 6 transitions per peptide. The peptide confidence was set to 91% (corresponding to <1% FDR by database search), and the SWATH peak group FDR threshold was set to 1%. Subsequently, peptides were selected manually to ensure that all peptides were fully tryptic (no missed cleavages) and contained no unusual modifications (oxidation of Met and pyroglutamination of N-terminal Gln were allowed). The fragment ions used for peak area extraction were manually curated to eliminate potential interferences and ensure the correct peak was selected. A retention time width of 5 min was used with a 75 ppm extracted ion chromatogram (XIC) width. The peak areas were exported to MarkerView software (version 1.3.1) for preliminary data quality checks and exported as a data frame for further statistical analysis.

### Statistical Analyses

Total fatty acids, starch, β-glucan, and triacylglycerol (TAG) content were examined to test the hypothesis that the nutrient content of BC-, CD-, and BD-mutant lines was different from wild-type (WT) with the null hypothesis as no difference. This was tested using ANOVA followed by Tukey’s test using the R statistical computing environment with differences with a corresponding *p* < 0.05 reported as significant.

Proteome analytics were performed with SIMCA (Sartorius Stedim Biotech; version 15.0), MetaboAnalyst (Xia et al., 2009), using the set of Poaceae proteins downloaded from the UniProt database (2018/03; 1,605,728 sequences). Data were pre-treated by log10 transformation and mean centring prior to analysis. Unsupervised analysis using Principal Component Analysis (PCA) was initially performed on SIMCA software to reveal any outliers and to assess any groupings or trends in the dataset. The pathway enrichment analysis was calculated using the Benjamini-Hochberg false discovery rate-adjusted p-value < 0.05 and fold change >2. Mean log2 ratios of biological triplicates and the corresponding p-values (*p* ≤ 0.05) were visualised using volcano plots. One-way ANOVA with multiple comparisons correction was used to compare the median of five experimental groups depicted as violin plots using Biovinci v 1.1.4) (BioTutoring Inc., San Diego, California, United States). Gene Ontology (GO) analysis was performed in BLAST2GO (OmicsBox, Biobam) (Conesa et al., 2005).

### Amino Acid Composition Change Estimation

The overall bound amino acid composition in DM lines were estimated by using the Biopython collection of tools. In this regard, sets of proteins with significantly higher or lower abundance in the mutant lines (*cf* WT lines) were provided to the ProtParam (Gasteiger et al., 2005) tool to determine percent amino acid composition for each protein within a set. The mean percent of each amino acid was then determined, and the composition of higher abundance proteins divided by the composition of lower abundance proteins. This measure is intended to provide an indication of the amino acid compositional change induced by gene knockout but does not account for protein abundance.

To investigate changes in the protein-bound amino acid composition at a protein level the individual amino acid contents were normalised against the Viridiplantae protein dataset using the Protein Report tool in CLC Genomics Workbench v12.1 (Qiagen, Aarhus, Denmark). Proteins with a lysine content of at least 1.5 times higher than the plant background data set were considered as lysine-rich proteins.

### Chromosome Mapping of the Identified Proteins to the Barley Reference Genome

UniProtKB annotation of proteins with significant fold change values in any of the wild type – DM or DM – DM comparisons were reference against the barley reference genome protein IDs retrieved from EnsemblPlants (International Barley Genome Sequencing Consortium, Mayer et al., 2012). Proteins without a barley reference genome protein ID were mapped to the translated and annotated barley gene models (Hordeum_vulgare_IBSC_v2_pepall) using BLASTp (International Barley Genome Sequencing Consortium, Mayer et al., 2012). Chromosomal location and exact position of the mapped hits were collected and used to annotate the Circos plots (Yiming et al., 2018). Detailed methods for chromosomal mapping and protein-protein interaction maps were given in Supplementary File 1.

### Ternary Plot Analysis

Protein abundance patterns in the three DMs compared to the parent lines (single-SMs and wild-type) were plotted using Ternary plot.^1^ Normalised peak abundance values were used to calculate the plot parameters. Rdist was used to calculate Euclidean distances of proteins from protein abundance bias categories as described by Ramírez-González et al. (2018).

### Protein-Protein Co-abundance Network Analysis

The combined set of proteins showing significant changes in any of the WT-DM or DM-DM comparisons were used to build a protein-protein interaction network in Cytoscape v 3.7.2 (Shannon et al., 2003). The network was built using the ExpressionCorrelation plugin with a 0.95 correlation value cut off. The cluster ID for proteins showing similar abundance values as defined in the hierarchical clustering was used to annotate the network and to reveal relationships between the different protein groups. NetworkAnalyzer was used to define the network measures (Assenov et al., 2008).

### Promoter Motif Analysis

Non-coding sequences (1,000 base pairs long 5’-end) were extracted from the barley reference genome assembly and used for a promoter motif search analysis of BPBF transcription factor binding sites (International Barley Genome Sequencing Consortium, Mayer et al., 2012). Prolamin box (TGTAAAG and TGTAAAGT), Pyrimidine box (CTTTT), GA-MYB (AACA), GA (TAACAAA) motifs have been annotated with 100% sequence identity using CLC Genomics Workbench v12.1. The obtained annotation pattern was visualised using UpsetR in R/Shiny package Intervene (Khan and Mathelier, 2017).

## Results

### Phenotype Measurements Across Hordein-Mutant Lines

**FIGURE 1:**
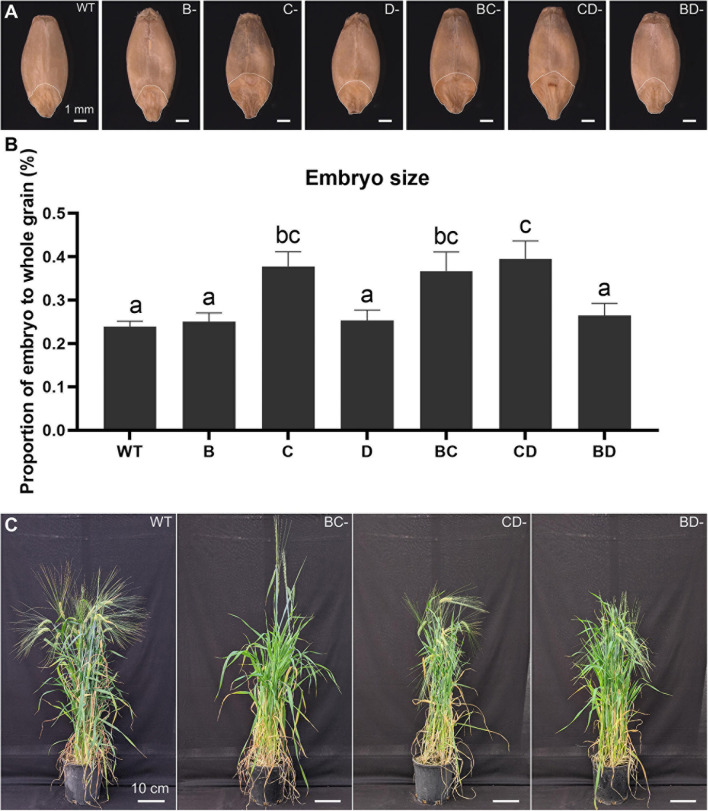
Plant phenotypes and embryo size measurement across the WT, single- and double-mutant lines. **(A)** Grain morphologies of WT, B-, C-, D-hordein single-mutant lines and BC-, CD-, and BD-hordein double-mutant lines. The region on the grain outlined with a white line represents the embryo. Scale bar = 1 mm. **(B)** Embryo size comparison presented as mean ± standard deviation. Different letters indicate that the mean in each bar is significantly different (*p* < 0.05) using ANOVA, Tukey’s HSD. **(C)** Plant morphologies of WT, BC-, CD-, and BD-hordein double-mutant lines. Scale bar = 10 cm.

Previous studies have shown that lines containing the Lys3 locus have larger embryos (Tallberg, 1977; Deggerdal et al., 1986; Cook et al., 2018). The embryo in the CD-mutant was the largest (65.6% increase), followed by BC-mutant (53.6% increase), while the BD-mutant was not significantly different from WT (Figure 1).

To measure the nutritional changes across the mutants, total fatty acid, TAG, starch content, and β-glucan were studied in detail. The CD-mutant line showed the substantial increase in total fatty acid, with an 82.2% increase as compared to the WT, followed by the BC-mutant with 67.6% increase and no significant change in the BD-mutant (Table 1). Triacylglycerol is one of the major components in the total lipid of barley caryopsis. To investigate how the change in total fatty acid content can affect the lipid composition in the DM lines, the total TAG level was measured across all lines. The highest increase in total TAG content was observed in the CD-mutant (127.3% increase), followed by the BC-mutant (110.1% increase); however, no significant change was observed in the BD-mutant (Table 1).

**TABLE 1:** Total content of fatty acid, TAG, starch and β-glucan of WT- and double-mutant lines.

Sample	Total fatty acid	Total TAG	Total starch content	Total β-glucan
WT	3.15(± 0.07)	1.39(± 0.20)	51.65(± 0.96)	3.29(± 0.04)
B-mutant	3.12(± 0.04)	1.47(± 0.27)	45.02(± 2.20)**	2.54(± 0.05)**
C-mutant	5.76(± 0.38)**	3.22(± 0.32)**	38.82(± 0.83)**	1.20(± 0.04)**
D-mutant	2.76(± 0.07)	1.26(± 0.18)	49.16(± 2.84)	3.36(± 0.07)
BC-mutant	5.28(± 0.04)**	2.92(± 0.35)**	38.42(± 2.60)**	1.40(± 0.10)**
CD-mutant	5.74(± 0.53)**	3.16(± 0.15)**	35.64(± 0.90)**	1.24(± 0.11)**
BD-mutant	3.47(± 0.23)	1.57(± 0.12)	42.95(± 0.90)**	2.09(± 0.07)**

All the measurement unit for fatty acid, TAG, total starch and beta-glucan is in g/100g (Dry Weight). Mean value (*n* = 3); ** double asterisks represent significant difference (*p* < 0.05) with wild-type by one-way ANOVA and Tukey’s HSD test. Tukey’s HSD (honestly significant difference) test was used to test the hypothesis that the nutrient content of null barley line was different from wild-type with the null hypothesis as no difference (Kirk, 1969; Tukey, unpublished). Statistical analyses were conducted using R and all the results reported as significant were those with significance level of *p* < 0.05 (R Core Team, 2013).

All three DM lines showed significant decreases in total starch content. The CD-mutant had a 31.0% reduction in total starch content, which was the highest among the three DM lines, followed by BC-mutant with a 25.6% reduction and a 16.9% reduction of total starch content in the BD-mutant (Table 1). The β-glucan content analysis was reduced in all three double-mutant lines. The highest reduction in β-glucan content was observed in the CD-mutant with a 61.3% reduction, followed by the BC-mutant with a 56.3% reduction and the BD-mutant with a 34.7% reduction (Table 1).

### Establishing a Proteomic Map of the Hordein Double-Mutant Lines

To investigate the extent to which the combination of two single hordein mutants altered the proteome of the three barley DM lines, DIA-based proteome measurement was used to detect and quantify proteins across the barley lines. To this end, information-dependent acquisition enabled the identification of 2,446 proteins, which were used in the construction of a peptide ion library. The ion library was manually curated at the peptide level to remove sequences with missed cleavages and/or variable modifications such as deamidation to improve quantitative accuracy in the resultant data frame. A total of 6,138 unique peptide sequences were quantified (representing 1,907 unique proteoforms). Subsequently, the resultant data (ion, peptide and protein) were exported to MarkerView software (SCIEX) to perform an initial quality check. No unexpected outliers or stratification was observed between samples at ion, peptide and protein resolutions. As a result, the protein level data matrix was selected for further statistical and functional analyses.

**FIGURE 2:**
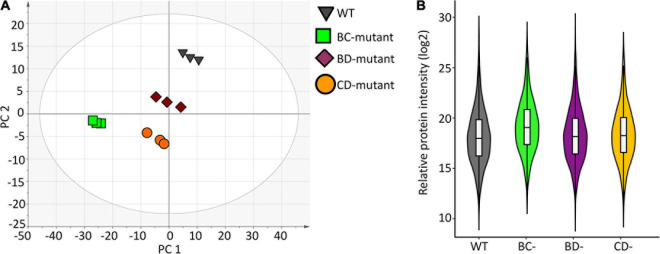
Data structure and variance within the proteome measurement of WT and double-mutant lines. **(A)** PCA scores plot shows separation due to protein abundance variation for WT and double-mutant lines. Each symbol represents one replicate from a barley line. **(B)** Violin plot depicting protein variance from wild-type (WT) and three double mutant lines (*n* = 3).

A high-level assessment of proteomic similarities and differences across the barley lines was accomplished using principal component analysis (Figure 2A). The WT replicates clustered together in principal component 1 (PC1) and explain the largest variation of the *x*-dimension. Overall, the three-component PCA model explains ∼72% of the total variance within the dataset where 54% and 13% of the variance were modelled to PC1 and PC2, respectively.

Violin plots were generated using the protein measurements for the three biological replicates originating from individual experimental groups to evaluate the overall variation (Figure 2B). No significant differences were observed between WT and the BD-mutant line. However, significant protein abundance variation was observed between WT and the BC-mutant (*p* < 0.009) and the CD-mutant (*p* < 0.02) lines. Protein quantitation using the Bradford protein assay supports the variations observed in the SWATH-MS data across the experimental groups. The Bradford protein estimates the wild-type as 4.2 mg/mL of protein in extracts from a 100 mg flour equivalent (per mL of solvent). In the DM samples, the total amount of proteins presents 3.7, 2.5 and 2.7 mg/mL of proteins in the BC-, CD-, and BD-mutant lines, respectively. Notably, the protein content in the DM lines were >1.5 fold increased in comparison to SM lines (Supplementary Table 1). For instance, the protein content in the B- and C-mutants were 2.1 and 1.4 mg/mL, respectively; whilst upon combining the two recessive lines the protein content was 3.7 mg/mL.

### Altered Proteome in DMs With *lys3*-Mutant Background Reveals Repression and Compensation Mechanisms

**FIGURE 3:**
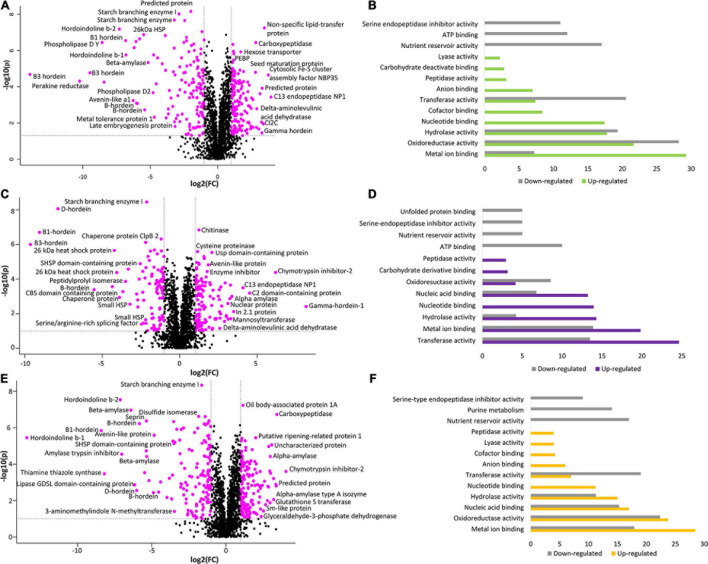
The comparison between WT and double-mutant lines illustrates the alteration of protein abundances and their associated functional classes. **(A)** Volcano plot of fold-change vs. p-value for comparison of BC-mutant and WT lines. **(B)** GO analyses (molecular function) performed on perturbed proteins: increased (green) or decreased (grey) in the BC-mutant line relative to WT. **(C)** Volcano plot of fold-change vs. p-value for comparison of BD-mutant and WT lines. **(D)** GO analyses (molecular function) performed on perturbed proteins: increased (purple) or decreased (grey) in the BD-mutant line relative to WT. **(E)** Volcano plot of fold-change vs. p-value for comparison of CD-mutant and WT lines. **(F)** GO analyses (molecular function) performed on perturbed proteins: increased (orange) or decreased (grey) in the CD-mutant line relative to WT. The pink points in the volcano plots represent proteins with significant perturbation (*p* < 0.05; FC < 0.5 or FC > 2.0).

To further explore the proteome measurements, a volcano plot was generated to compare the protein abundance differences between WT and BC-mutant lines. The results indicate that there was alteration in 296 (15.5%) proteins wherein 151 (7.9%) proteins were more abundant in the BC-mutant line whilst 145 (7.6%) proteins were less abundant within the BC-mutant line (Figure 3A).

To obtain functional insights into the BC-mutant line proteome changes, the protein accessions that yielded identifications to non-barley Poaceae proteins, due to the incomplete nature of the public barley protein databases, were subjected to homology searching using BLASTp to find *H. vulgare* orthologs. The GO-based analysis revealed that molecular functions including metal ion binding, oxidoreductase activity, and enzymatic activities were increased in the BC-mutant lines (Figure 3B). The molecular functions associated with the down regulated proteins from the BC-mutant lines included oxidoreductase activities, enzyme activities and nutrient reservoir activity (Figure 3B).

The comparison between the BD-mutant line with the WT identified the perturbation of 200 (10.5%) proteins. Within these proteins, 113 (5.9%) were significantly more abundant in the BD-mutant lines whilst 87 (4.6%) proteins were less abundant (Figure 3C). Proteins involved in catalytic activities such as transferase, hydrolase and oxidoreductase molecular functions were more abundant (Figure 3D). As expected, proteins involved in nutrient reservoir activities were less abundant along with the serine-endopeptidase inhibitor activity (Figure 3D).

The comparison between WT and CD-mutant lines reveals the alteration of 274 (14.4%) proteins; wherein 138 (7.2%) proteins were more abundant and 136 (7.1%) proteins were less abundant (Figure 3E). In accordance with the other DM lines, enzymatic activities such as oxidoreductase, hydrolase, transferase, lyase and peptidase activities were more abundant along with metal ion and nucleic acid-binding (Figure 3F). On the other hand, proteins involved in nutrient reservoir function and serine endopeptidase inhibitors, primary metabolic pathways and enzymatic activities were less abundant (Figure 3F).

### Pairwise Comparison Between WT and DM Lines Reveals the Link Between Parent and DM Lines

**FIGURE 4:**
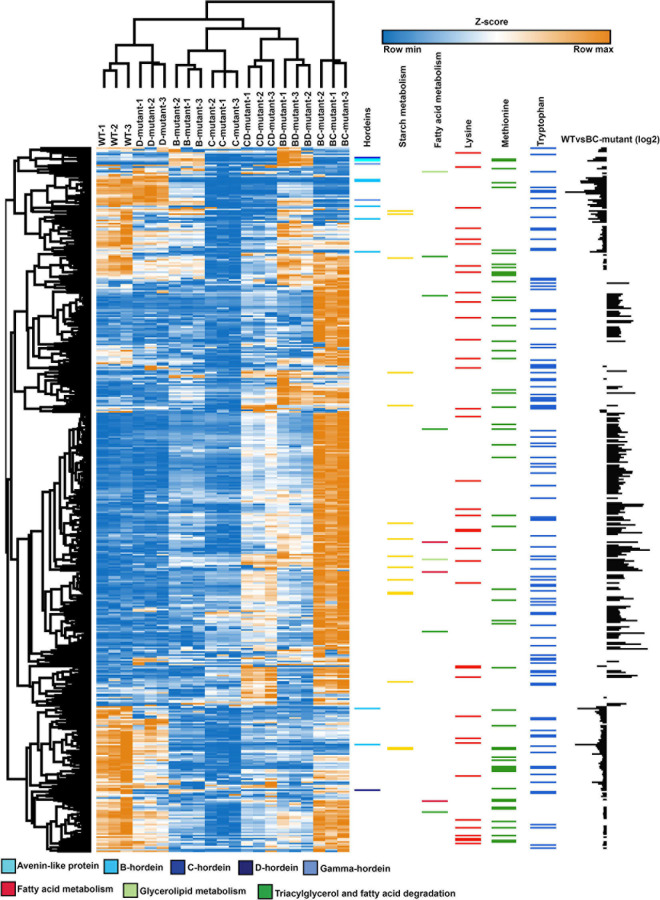
Abundance profile of the chromosome mapped and differentially abundant proteins across the WT, single- and double-mutant lines. Heatmap showing the relative abundance levels (Z-scored) of proteins across the single and double-mutant barley lines in comparison to WT. Colour bars represent the abundance of hordeins, starch and fatty acid metabolism, lysine, methionine, and tryptophan-rich proteins. The individual amino acid contents were calculated using the Protein Report tool in CLC Genomics Workbench. The bar chart (last column) illustrates the log2 abundance of differentially abundant proteins in the WT and BC-mutant lines.

A heatmap was generated from the differentially abundant proteins identified from pairwise comparisons between WT and DM lines to visualise the link between single and DM lines. The SM proteome abundance profiles were also included in the heatmap to allow contrast to the parent lines (Figure 4) (Bose et al., 2020). Hierarchical clustering shows the grouping of the biological replicates and that the D-hordein mutant line is closely linked to the WT, as D-hordein proportionately represents just 1–2% of total hordein content resulting in a minimal perturbation to the proteome.

To further explore proteome, co-regulation and phenotype perturbation, the differentially abundant proteins were functionally annotated and plotted as heatmaps (Figure 4 and Supplementary Table 3). The comparison between the WT and BC-mutant line was used as an example as B- and C-hordeins represent >95% of total hordein content in barley grain and thus the BC-mutant is expected to have substantial impact on the proteome compared to the remaining DM lines. Figure 4 highlights the abundance of hordeins, fatty acid metabolism and starch metabolism-associated proteins along with lysine, methionine and tryptophan-rich proteins. The abundance of lysine-rich proteins was increased in the BC-mutant line whilst the proteins associated with nutrient reservoir functions were decreased. Proteins associated with fatty acid synthesis were more abundant (Figure 4). The final column illustrates the relative changes in protein abundance between the WT and BC-mutant lines, thereby revealing that the nutrient reservoir activity-related proteins were less abundant in the BC-mutant line (Figure 4). Proteins sharing similar abundance profiles among the DMs and their parents clustered into 12 modules (Supplementary File 1). Module 1 is composed of 13 proteins and is enriched in C- and γ-hordeins and avenin-like proteins (ALPs). Additionally, B-hordeins are clustered in module 4 along with 12S seed storage globulin while D-hordein are in module 12. Module 4 includes 24 proteins that are either BD-dominant or balanced when comparing the three DMs. Proteins involved in starch and carbohydrate metabolism are clustered in module 4, 5, and 6 encompassing 24, 24, and 51 proteins, respectively. The largest module, module 9 is composed of 161 proteins that are enriched under energy and carbohydrate metabolism functions. Module 11 is enriched in proteins related to protein processing in the endoplasmic reticulum with the abundance of these proteins mostly balanced between the DM and WT.

A supervised Orthogonal Projections to Latent Structures Discriminant Analysis (OPLS-DA) model was built to identify how the SM parent lines contribute to the alteration of DM lines. Therein, the WT, B-, and C-mutant lines stratified in class 1 space while the BC-mutant line stratified in the class 2 space. Next, the analysis of VIP (Variable Importance in Projection) plot allowed the identification of the top 100 most influential proteins (VIP > 1.2), which were altered in their abundance in the BC-mutant line when compared to WT, B-, and C-mutant lines (Supplementary Figure 1 and Supplementary Table 3). To determine the magnitude of proteome perturbation, the mutant lines were compared with the WT (Supplementary Figure 1). The proteins with perturbed abundance in the BC-mutant line was highly concordant with the changes observed in the parent B- and C-hordein mutant lines (Bose et al., 2020). Additionally, the *lys3* mutant, *i.e.*, removal of C-hordein, had a dominant influence on the proteome in the BC-mutant line (Supplementary Figure 1).

### Alteration of Amino Acid Compositions in Barley DM Lines

To measure the amino acid composition changes between WT and DM lines, the lists of differentially abundant proteins were collected from the pairwise comparative analysis (Supplementary Table 3). One essential (His) and three non-essential amino acids (Ala, Gly, and Tyr) were significantly increased in the DM lines. As expected, glutamine and proline were significantly decreased by up to 47% and 15% in the prolamin-depleted BC- and CD-mutant lines, respectively, but not in the BD-mutant line. Interestingly, essential amino acids such as lysine were found to be most abundant in the BC-mutant line, while proline was significantly decreased (Supplementary Figure 2). In accordance with the current finding, previous study has also shown that the incorporation of mutant lines significantly increased the free amino acids (Tanner et al., 2015).

**FIGURE 5:**
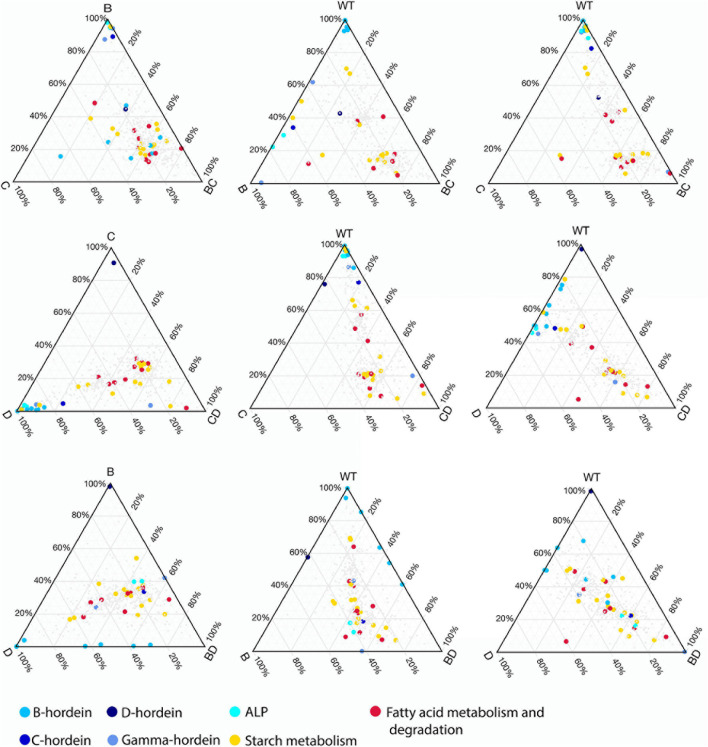
Changes in relative abundance patterns in the double-mutant and parent lines. Ternary plots were constructed to highlight relative abundance differences between DMs, SMs, and WT. For each DM three plots are presented representing DM and SM relationships followed by DM, WT relationships combined with the presence of one of the SM parents. The colours highlight the different hordeins, starch metabolism, fatty acid metabolism and degradation associated proteins.

Ternary plots were used to explore the abundance patterns of proteins in the DMs and their relationship with each other or their parent mutant lines across the three double-mutant lines (Figure 5 and Supplementary Figure 3). Two of the B-hordeins (I6TMW0 and A0A0K2GRQ1) have a balanced abundance when the three DMs are compared (Supplementary Figure 3A), while other B-hordeins as well as gamma- and C-hordeins show a relative enrichment in the BD-mutant compared to the BC- and CD-mutant lines. However, these proteins show a balanced abundance when BD- is compared to D- and WT indicating that B-hordeins with a higher abundance value in the BD-mutant line might be inherited from the D-hordein mutant parent (Figure 5). Comparing the relative distribution of the fatty acid metabolism-related proteins in the three DMs compared to their parents, most of the fatty acid metabolism-related proteins show a balanced abundance in the DMs with a D-hordein mutant in the background. However, these proteins are relatively enriched in the BC-mutant when compared to the WT or any of the single mutant parents. Comparing the relative abundance of starch metabolism related proteins, they are depleted in the DMs with the C-hordein mutant parent in the background; however, they show a balanced abundance if B- or D-hordein mutant lines are present.

### Altered Proteins From the Double-Mutant Lines Indicate Orchestrated Changes in Nutrient, Starch, and Fatty Acid Metabolism

To elucidate the effect of protein alteration at the chromosome level, hordeins and proteins showing significant changes in abundance were mapped to the Morex barley reference genome (International Barley Genome Sequencing Consortium, Mayer et al., 2012). Gamma-, B-, and C-hordein sequences were mapped to the short arm of chromosome 1H and clustered in five loci, representing two B-hordein, two C-hordein and a gamma-hordein loci (Supplementary Figures 3B,C). Detailed annotation of mapped protein sequences including their chromosomal position is presented in Supplementary Table 2.

**FIGURE 6:**
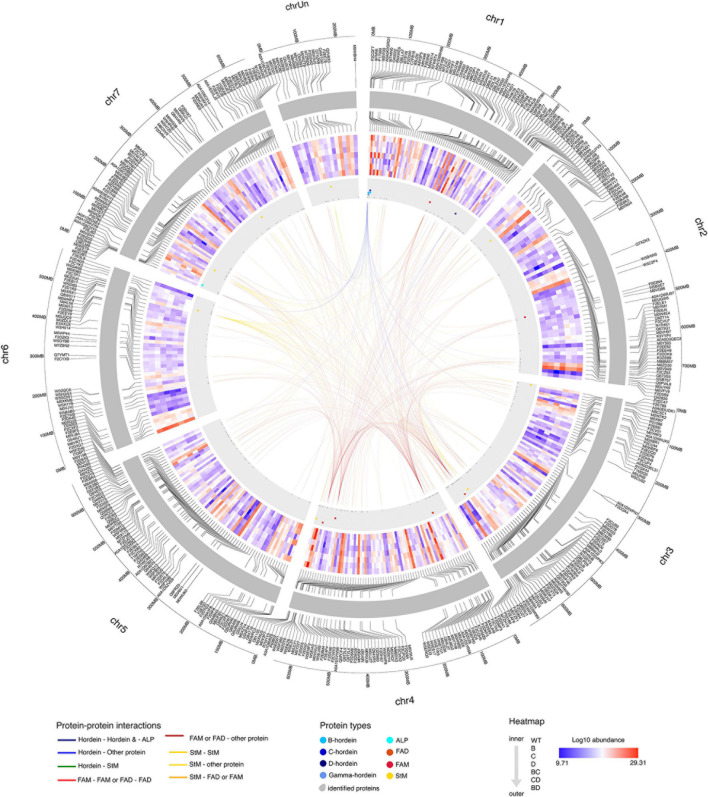
Protein-protein interaction patterns of highlighted hordeins, fatty acid metabolism and degradation and starch metabolism associated proteins. Chromosomes are visualised in the outer circle as grey bands. Mapped proteins showing significant fold-changes compared to the WT are labelled with accession numbers. Log2 protein abundance values are presented in the heat map section. The direction of samples indicated from the inner to the outer circle as WT, single mutants B-, C-, and D-, double-mutants BC-, CD-, and BD-, respectively. The position of hordeins and the highly related avenin-like proteins are shown as blue dots in the grey annotation band, fatty acid metabolism and fatty acid degradation associated proteins are highlighted in red and deep red colours, while orange dots represent proteins related to starch metabolism. Links between chromosome regions represent potential interactions. Only correlations above 0.95 (strong positive) and below -0.95 (strong negative) are highlighted. Interactions are coloured to indicate the interactor types.

B-hordeins located in the B hordein locus 1 (Supplementary Figures 3B,C and Supplementary Table 2) share similar abundance patterns with starch branching enzyme I mapped to the unassigned chromosome (Chr Un), protein folding-related heat-shock proteins and chaperones from chromosomes 1H, 5H, 4H, and 6H (Figure 6). Two of the B-hordeins, R9XWE6 and C7FB16 were found in higher amounts in the D-hordein mutant compared to the B- and C-hordein mutant lines, and were enriched in the BD-mutant compared to the BC- and CD-mutants, sharing opposite abundance patterns to energy metabolism proteins (e.g., M0WGK7, M8C3P1, M0Y565). The C-hordein I6TEV8 shared highly similar abundance patterns to ALPs from chromosome 7H and the gamma-hordein in 1H. The C-hordein was higher in abundance in the BD-mutant line when compared to the other DMs and was a dominant protein in the WT (Figure 6).

Proteins with functions in fatty acid degradation shared similar abundance trends with many of the lysine-rich proteins such as LEA proteins (C9ELM9, B5TWD1, F2DZK3) from chromosomes 7H, 1H and 6H. Proteins linked to fatty acid metabolism (F2CWX3 and W5BXE7) were associated with proteins enriched in lysine, methionine or threonine, such as disease defence proteins and seed maturation-related proteins mainly located at chromosomes 1H, 3H and 5H.

Starch metabolism associated links have been identified between chromosomes 3H, 4H, 6H and 7H. Among the proteins with highly similar abundance patterns there were 25 lysine-rich proteins. Some of these, like a seed maturation-related dehydrin (Q40043), an energy metabolism related glyceraldehyde-3-phosphate dehydrogenase (M0Y565) and a protein with unknown function (M0YTG0) show a strong negative correlation with the B-hordeins enriched in DMs with a D-hordein mutant origin, while a calreticulin (M0V198), a HSP90 protein (M0 × 173), a chaperone and a calcium-binding protein were positively correlated.

To further investigate the co-abundance relationships between lysine-rich proteins and hordeins and other cysteine-rich prolamin superfamily members, 1,000 bp non-coding promoter regions of the coding genes of mapped proteins were searched for transcription factor binding sites characteristic of BPBF (Prolamin-box with a DOF core motif, Pyrimidine-box and GAMyb motif). Twenty-three of the mapped lysine-rich protein coding genes contain both the pyrimidine box and a DOF core motif in their sequence within the first 1,000 nucleotide upstream of the start codon (Supplementary Figure 4 and Supplementary Table 2). Comparing the promoter motif composition of proteins from the various modules shows potential negative interactions between proteins with Pyr-GAMyb and Pyr-Pbox or Pyr-Pbox and Pyr-Pbox-GAMyb TFBS containing promoter regions. While interacting partners from the energy, fatty acid and carbohydrate metabolism module 6, 9 and 10 that show similar positive abundance trends mostly have Pyr-GAMyb and Pyr-GAMyb or Pyr-Pbox-GAMyb TFBSs. Modules enriched in storage proteins (module 1, 3, and 4) and ER-acting, folding and ripening-related proteins are characterised with the presence of Pbox motifs either in a combination with Pyr-box or with Pyr-box and GAMyb motifs (Supplementary Table 2).

## Discussion

The temporal and spatial regulation of cereal storage protein synthesis is orchestrated by precise mechanisms primarily at the transcription level. High resolution genome sequences and gene model annotations help to understand, utilise and process the genomic information characteristic of seed development and maturation programmes. The suppression of three hordein subclasses B, C, and D within the barley SM lines led to multiple repression and compensation events. These events are further evident in the double-recessive lines where the compensation events more clearly depend on their parent lines (Figures 5, 6 and Supplementary Figure 1). Additionally, the incorporation of mutant lines significantly affects the free amino acids (Tanner et al., 2015) and bound amino acids analysed in the present study (Supplementary Figure 2).

The positions of the hordein coding genes in chromosome 1H were determined using the available barley hordein sequences. In the Morex reference genome both the B-hordeins and the C-hordeins are clustered into two separate loci (Supplementary Figure 3B). Most of the B-hordein coding proteins are located in the B-hordein locus 1 (2.43 – 3.30 Mb), while some additional B-hordein coding genes were mapped to the 11.86 – 11.96 Mb region. In the B-hordein mutant line the γ-ray induced mutation deleted ∼85 kb of the storage protein enriched distal 1H end and eventually caused the depression in hordein accumulation (∼75%) in the grain endosperm (Shewry et al., 1980; Kreis et al., 1983). The B-hordeins from both B-hordein loci are significantly less abundant in the BC-mutant line compared to the WT while a slight compensation can be seen in some of the B-hordein abundance values both in the BC- and BD-mutant lines (Supplementary Figures 3A,B). This provides evidence that the proteins I6TMW0 and Q40022 might be inherited from the C and D single mutant parent in the BC- and BD-double mutant lines. Due to incomplete deletion of B-hordein locus, and the existence of a variety of hordein gene models mapped to the chromosome 1H (Supplementary Figure 3B), it is possible that the B-mutant line, Risø 56, carries additional hordein coding regions of which gene products were not detected herein. The remaining B-hordein-type proteins are evident in the B-mutant parent line where a hordein closely related to B-hordein subclasses was increased up to ∼8 fold (Bose et al., 2020) as well as two low molecular weight B-hordeins (C7FB16, R9XWE6) from the B-hordein locus 2 in the BD-double mutant line (Figure 5 and Supplementary Figure 3B). Notably, the deletion of the major B-hordein locus did not affect the C-hordein locus. As a result of B-hordein removal, the total abundance of C-hordein and γ-hordein were shown to increase, possibly to compensate for the loss of B-hordeins. Interestingly a clear positive interaction was detected between the abundance patterns of C-hordeins, γ-hordeins and some ALPs encoded on the short arm end of chromosome 7H (Supplementary Figure 3C). ALPs represent a protein subgroup within the prolamin superfamily that, similarly to gliadins, possess gliadin Pfam domains (PF13016) (Juhász et al., 2018). These proteins were reported to have both storage protein functions and are also involved in stress defence mechanisms (Zhang et al., 2018a, b).

In cereals, *lys3* encodes a transcription factor known as prolamin binding factor (PBF), located on the long arm of chromosome group 5 in wheat and 5H in barley, which express in the starchy endosperm, embryo and the aleurone during seed development and germination (Marzábal et al., 2008; Moehs et al., 2019). PBF acts as an activator or repressor through some known transcription factor binding site domains like the TGTAAG (Prolamin box and Pyrimidine-box (CCTTTT) (Shewry et al., 1995; Marzábal et al., 2008). In the developing seed PBF negatively regulates the GA-responsive production of thiol proteases, α-amylases, proteases, hydrolases and acts as an activator for the prolamin genes as well as genes involved in starch accumulation. PBF triggers storage protein and starch accumulation primarily by activating genes through the Prolamin-box, while in embryo-related genes and especially during germination it relates to gibberellic acid (GA) signalling and acts through the Pyrimidine box. The Pyrimidine box is usually part of the GARC triad (GA responsive complex of GAMyb + PBF + GA response factors) and is enriched in aleurone- and endosperm-related genes (Mena et al., 2002). The *lys3* mutant Risø 1508 has regulatory impact on a wider range of biochemical processes in addition to hordein synthesis (Orman-Ligeza et al., 2020). Indeed, the decrease in lysine-poor storage protein accumulation and starch content in the grains of *lys3* mutant plants lead to more lysine-rich proteins and free lysine (Ingversen et al., 1973). Generally, this loss of PBF TF activity is concomitant with an above average embryo size (Cook et al., 2018). Herein, stereomicroscope measurements show a significantly higher embryo size in the BC- and CD-double mutants providing evidence for the presence of an active PBF gene in the BD-mutant line that show similar embryo size to the WT (Figure 1).

Notably, an impact on embryo size was previously observed for the parental hordein SM lines used in this study (Bose et al., 2020). Herein, the double-mutant lines display repression of B- and C-hordeins resulting in a shift to an increased proportion of lysine-rich proteins such as seed folding and maturation related proteins, including 60S ribosomal proteins, LEA proteins, chymotrypsin inhibitors and serpins (Figure 3 and Supplementary Figure 1). In maize, the overexpression of elongation factor 1α (EF 1α) in the endosperm regulates the higher production of lysine-rich proteins (Habben et al., 1995). Similarly, here, a 60S acidic ribosomal protein with a translation elongation activity (M0XWV5) was measured to be >5-fold higher in the BC-mutant line in comparison to WT (Supplementary Table 3). This protein co-clustered with the energy metabolism and fatty acid metabolism associated proteins in module 9. In the endosperm, EF 1α can be found in the protein bodies where they have been associated with the cytoskeleton, bind to the endoplasmic reticulum, couple with the mitotic apparatus and several microtubules (Durso and Cyr, 1994). The cytoskeleton plays a key role in storage protein synthesis in cereal grains through their association with the rough endoplasmic reticulum membrane surrounding the protein bodies (Shewry et al., 1995; Bose et al., 2020). Thus, it is possible that the EF 1α concentration provides an index of a complex group of proteins making up the cytoskeleton that helps to redistribute nitrogen away from the normal sink in order to increase non-storage proteins.

Previous morphological and phenological studies have shown that the *lys3* mutation influences aleurone cell size, starch granule formations, β-glucan content, seed germination rate and fatty acid content (Arruda et al., 1978; Deggerdal et al., 1986; Christensen and Scheller, 2012; Moehs et al., 2019; Orman-Ligeza et al., 2020). In this study, all DM lines displayed a reduced total starch content. In the BC- and CD-mutant lines, the large embryo phenotype results in a corresponding reduction of starchy endosperm. As this is the major storage tissue for starch in seed (Duffus and Cochrane, 1993; Radchuk et al., 2009), the reduction in this compartment likely corresponds to the reduced total starch content of the seed. However, the embryo size of the BD-mutant line is unchanged, therefore, the total starch content may be affected by factors other than the embryo starchy endosperm proportion. Therefore, it is highly likely that the combination of D-hordein mutant with the B- and C-hordein mutant lines activates the starch and storage protein accumulation or regulates the starch and B- and C-hordein contents.

In barley *lys5* mutants, the starch synthesis was reduced due to the lesions of *Hv.Nst1* genes located on chromosome 6, which encodes a plastidial ADP-Glc transporter (Patron et al., 2004). Herein, an ADP-Glc transporter protein (Q6E5A5) located in chromosome 7H was shown to have no significant changes in the single- and double-mutant lines (Supplementary Table 2). Although *lys5* mutants have decreased starch content, the major component of cell wall (1,3;1,4)-β-D-glucan was increased due to higher concentration of cytosolic UDP-Glc (Rudi et al., 2006; Christensen and Scheller, 2012). Yet, in the *lys3* mutants the (1,3;1,4)-β-D-glucan content was reduced 1,000-fold due to the repression of *Cellulose Synthase-Like* (CSL) *F6* transcript throughout the endosperm development period (Christensen and Scheller, 2012). In this study, the β-glucan [(1,3;1,4)-β-D-glucan] content in BC- and CD-mutant lines was reduced. This indicates that *lys3* transcriptional regulation from C-hordein mutant lines can regulate *CSLF6* expression in double mutant lines to reduce the β-glucan content. Although CSLF6 was not detected herein, other genes related to β-glucan metabolism were perturbed. Q8S3U1 is a β-1,3-glucanase II and Q9XEI3 a β-D-glucan exohydrolase isoenzyme, both functioning in β-glucan degradation, were significantly increased in all three double-mutants. These β-glucan-related proteins show negative concordance with a range of fatty acid metabolism and lysine-rich proteins in the DMs with a *lys3* mutant parent. Of interest, the abundance pattern of these β-glucan metabolism-associated proteins is similar in all three double-mutants with the highest abundance in the BD-mutant line, which cannot be explained by the mutation in the PBF gene. Quantitative trait loci linked to β-glucan content in wheat were found on chromosomes 1A, 2A, 2B, 5B, and 7A (Marcotuli et al., 2016). Among these, a glycosyl transferase is located in the short arm end of chromosome 1H in a relatively close proximity to the hordein loci. It is therefore possible that this protein is inactive or suppressed in the mutant lines with B-mutant in the background resulting in a decrease of β-glucan content. Additionally, it may also possible that the *CSLF6* repression results from the hypermethylation of the upstream promoter region of the *lys3* mutants, as this promoter is shared among these genes.

Unlike starch, the total fatty acid (FA) and TAG content was increased in the BC- and CD-mutant lines and their abundance can be directly linked to the *lys3* mutant, *i.e.*, C-hordein-mutant line (Figure 1). In this barley *lys3* mutant, three intermediates of lipid metabolism, including 2-hydroxyheptanoate, 9,10-dihydroxy-octadecanoic acid methyl ester and dimethyl ester of threo-9,10-dihydroxy-octadecanedioic acid, were found to be more abundant (Khakimov et al., 2017). This mutant may use excess lysine to convert to acetyl-CoA and thus provide more precursors for fatty acid synthesis leading to increased abundance of FA and TAG. Additionally, seeds may use these excess reserves as an energy supply to exit dormancy and initiate germination. The *Lys* mutation also influences the accumulation of Lys-rich proteins in the BC and CD DMs; abundant protein functions were associated with protein translation, folding and seed maturation. The abundance of the *Lys-*rich *late* embryogenesis abundant (LEA) protein in the *Lys* mutants are known to provide desiccation tolerance and co-regulated with abscisic acid to extend the dormancy period (Tan and Irish, 2006; Angelovici et al., 2009). The higher abundance of lysine-rich LEA proteins and dehydrins combined with reduced TCA cycle metabolism offers some explanation regarding the low germination success and reduced growth vigour for *lys3* mutants.

## Conclusion

Cereals are lower in grain protein content and essential amino acids such as lysine compared to legumes and oilseeds. Selectively breeding cultivars with more desirable amino acid composition has been attempted in cereal grains to improve the nutritional quality for human and livestock consumption. Yet, the low agronomic yield, germination defects and technological challenges for cereals have presented barriers for the commercial uptake of these crops. Within the DM lines measured herein, the BC-mutant lines are more suitable for those suffering with celiac disease. The D-hordeins, homologs of the wheat high molecular weight glutenins, have only shown a mild immune response for the patients with HLA-DQ8 (Molberg et al., 2003) and even less immune-reactivity for patients with HLA-DQ2 alleles. The accumulation of HMW glutenins are not affected in wheat PBF mutant lines (Moehs et al., 2019), and similar results were obtained in the present study for D-hordeins. Therefore, the reduction in hordein content, improvement in amino acid composition, while concomitantly maintaining desirable agronomic and functional characteristics through this DM line, offers a promising step toward production of a grain with desirable health and commercial traits.

## Data Availability Statement

The original contributions presented in the study are publicly available. This data can be found here: The mass spectrometric DDA and DIA raw data and result files have been uploaded to the CSIRO public data access portal. Data can be accessed through: https://doi.org/10.25919/5d5c83eb2e6dc.

## Author Contributions

UB and AJ analysed the dataset and prepared the first draft. RY performed phenotype analysis. MBa annotated the proteomics dataset. KB prepared proteomics samples and reviewed the manuscript. MBl performed the plant breeding and flour sample preparation. JB analysed the amino acid profiles and reviewed the manuscript. MC acquired the LC-MS dataset. MC and CH conceived the project idea and critically reviewed the manuscript. All authors contributed to the article and approved the submitted version.

## Conflict of Interest

The authors declare that the research was conducted in the absence of any commercial or financial relationships that could be construed as a potential conflict of interest.

## Publisher’s Note

All claims expressed in this article are solely those of the authors and do not necessarily represent those of their affiliated organizations, or those of the publisher, the editors and the reviewers. Any product that may be evaluated in this article, or claim that may be made by its manufacturer, is not guaranteed or endorsed by the publisher.

## Footnotes

1
https://www.ternaryplot.com

## Supplementary Material

Supplementary Figure 1 Abundance profile of the 100 differentially abundant proteins across the WT, B-, C-, and BC-mutant lines. Heatmap showing the relative abundance levels (Z-scored) of significant altered proteins across the lines. Colour bars represent the abundance of proteins associated with GO functions such as fatty acid synthesis, nutrient reservoir and inhibitory activity. The bar charts (last three column) shows the log2 abundance of differentially abundant proteins in the WT and B-, C-, and BC-mutant lines.

Supplementary Figure 2 Protein bound amino acid composition changes between double-mutant and WT lines. Amino acid composition was determined for proteins significantly perturbed by hordein deletion and expressed as a fold-change for the composition of proteins that are more abundant in mutant lines divided by the composition of proteins that are more abundant in the WT. These fold change values were log2 transformed for symmetry in the heat map where darker red indicates higher abundance in the mutant line and darker blue indicates higher abundance in the WT. Essential amino acids are indicated by black row side colouring.

Supplementary Figure 3 **(A)** Ternary plot representing relative abundance comparison of the three DMs. Abundance bias categories highlighting DM dominant, DM suppressed and balanced abundance are presented. **(B)** Ternary plot highlighting relative abundance values of the analysed hordeins. Mapped hordein locus positions are also labelled. **(C)** Genomic location of barley hordeins indicate the presence of multiple B- and C- hordein loci on the short arm of chromosome 1H as identified using publicly available hordein sequences mapped to the barley reference genome. The position of identified genes is labeled by circles and is differentiated for each hordein type using the colours presented in the colour legend. The first C-hordein locus is located at 1.45 Mb and is composed of at two C-hordein genes. This locus is followed by the gamma-hordein locus at 1.75 Mb and is composed of at least of three gamma-hordein genes. The B hordein locus 1 spans between 2.52 and 3.25 Mb and includes 11 gene models. The second C-hordein locus covers a 200 kb region between 11 and 11.2 Mb and includes nine C-hordein gene models. This gene cluster is followed by further two B-hordein genes at 11.95 Mb. In the proteomic analyses of the double-mutants we have identified two hordein sequences from the gamma hordein locus, six hordein sequences in B-hordein locus 1, two sequences in the C-hordein locus 2 and two sequences in the B-hordein locus 2. The position of identified proteins were labelled in coloured dots.

Supplementary Figure 4 Enrichment of Prolamin box variants (TGTAAAGT, TGTAAAGG, TGAAAAGT, TGAAAAGG, TGTAAAG), DOF-core motif (A/TAAG) and Pyrimidine-box (CCTTTT) motifs.

Supplementary Table 1 Hordein content of WT and hordein-single and DM lines.

Supplementary Table 2 Detailed annotation of mapped protein sequences including their chromosomal position.

Supplementary Table 3 Differentially abundant proteins were functionally annotated and plotted as heatmaps.

Supplementary File 1 Detailed materials and methods for embryo size measurement, barley β-glucan analysis, total starch content analysis, total fatty acid, and total TAG content analysis and circus plot.

## References

[B1] Angelovici R. Fait A. Zhu X. Szymanski J. Feldmesser E. Fernie A. R. (2009). Deciphering transcriptional and metabolic networks associated with lysine metabolism during *Arabidopsis* seed development. *Plant Physiol.* 151 2058–2072. 10.1104/pp.109.145631 19783646 PMC2785976

[B2] AOAC (1995). Starch (total) in cereal products, amyloglucosidase—α−amylase method 996.11. *J. Assoc. Off. Anal. Chem.* 55–58.

[B3] Arruda P. Da Silva W. Teixeira J. (1978). Protein and free amino acids in a high lysine maize double mutant. *Phytochemistry* 17 1217–1218. 10.1016/s0031-9422(00)94558-8

[B4] Assenov Y. Ramírez F. Schelhorn S.-E. Lengauer T. Albrecht M. (2008). Computing topological parameters of biological networks. *Bioinformatics* 24 282–284. 10.1093/bioinformatics/btm554 18006545

[B5] Batterham E. S. (1992). Availability and utilization of amino acids for growing pigs. *Nutr. Res. Rev.* 5 1–18. 10.1079/nrr19920004 19094310

[B6] Bose U. Broadbent J. A. Byrne K. Blundell M. Howit C. A. Colgrave M. L. (2020). Proteome analysis of hordein-null barley lines reveals storage protein synthesis and compensation mechanisms. *J. Agric. Food Chem.* 68 5763–5775. 32374605 10.1021/acs.jafc.0c01410

[B7] Christensen U. Scheller H. V. (2012). Regulation of (1, 3; 1, 4)-β-d-glucan synthesis in developing endosperm of barley lys mutants. *J. Cereal Sci.* 55 69–76.

[B8] Colgrave M. L. Byrne K. Blundell M. Heidelberger S. Lane C. S. Tanner G. J. (2016). Comparing multiple reaction monitoring and sequential window acquisition of all theoretical mass spectra for the relative quantification of barley gluten in selectively bred barley lines. *Anal. Chem.* 88, 9127–9135. 10.1021/acs.analchem.6b02108 27533879

[B9] Conesa A. Götz S. García-Gómez J. M. Terol J. Talón M. Robles M. (2005). Blast2GO: a universal tool for annotation, visualization and analysis in functional genomics research. *Bioinformatics* 21 3674–3676. 10.1093/bioinformatics/bti610 16081474

[B10] Cook F. Hughes N. Nibau C. Orman-Ligeza B. Schatlowski N. Uauy C. (2018). Barley lys3 mutants are unique amongst shrunken-endosperm mutants in having abnormally large embryos. *J. Cereal Sci.* 82 16–24. 10.1016/j.jcs.2018.04.013 30245543 PMC6142819

[B11] Deggerdal A. Klemsdal S. Olsen O. A. (1986). The effect of the high lysine genes of the barley mutants Risø 1508 and 527 on embryo development. *Physiol. Plant* 68 410–418. 10.1111/j.1399-3054.1986.tb03374.x

[B12] Doll H. (1973). Inheritance of the high-lysine character of a barley mutant. *Hereditas* 74 293–294. 10.1111/j.1601-5223.1973.tb01131.x 4755819

[B13] Duffus C. Cochrane M. (1993). “Formation of the barley grain-morphology, physiology, and biochemistry,” in *Barley: Chemistry and Technology*, eds Macgregor A. W. Bhatty R. S. (Madison, WI: American Association of Cereal Chemists), 31–72.

[B14] Durso N. A. Cyr R. J. (1994). A calmodulin-sensitive interaction between microtubules and a higher plant homolog of elongation factor-1 alpha. *Plant Cell* 6 893–905. 10.1105/tpc.6.6.893 8061523 PMC160487

[B15] Gabert V. M. Brunsgaard G. Eggum B. O. Jensen J. (1995). Protein quality and digestibility of new high-lysine barley varieties in growing rats. *Plant Foods Hum. Nutr.* 48 169–179. 10.1007/bf01088313 8837876

[B16] Gasteiger E. Hoogland C. Gattiker A. Wilkins M. R. Appel R. D. Bairoch A. (2005). “Protein identification and analysis tools on the ExPASy server,” in *The Proteomics Protocols Handbook*, ed. Walker J. M. (Totowa, NJ: Humana Press), 571–607. 10.1385/1-59259-890-0:571

[B17] Gillet L. C. Navarro P. Tate S. Röst H. Selevsek N. Reiter L. (2012). Targeted data extraction of the MS/MS spectra generated by data-independent acquisition: a new concept for consistent and accurate proteome analysis. *Mol. Cell. Proteomics* 11 O111.016717. 10.1074/mcp.O111.016717 PMC3433915 22261725

[B18] Habben J. E. Moro G. L. Hunter B. G. Hamaker B. R. Larkins B. A. (1995). Elongation factor 1 alpha concentration is highly correlated with the lysine content of maize endosperm. *Proc. Natl. Acad. Sci. U.S.A.* 92 8640–8644. 10.1073/pnas.92.19.8640 7567989 PMC41022

[B19] Hansen M. Lange M. Friis C. Dionisio G. Holm P. B. Vincze E. (2007). Antisense-mediated suppression of C-hordein biosynthesis in the barley grain results in correlated changes in the transcriptome, protein profile, and amino acid composition. *J. Exp. Bot.* 58 3987–3995. 10.1093/jxb/erm254 18162630

[B20] Howard T. P. Fahy B. Craggs A. Mumford R. Leigh F. Howell P. (2012). Barley mutants with low rates of endosperm starch synthesis have low grain dormancy and high susceptibility to preharvest sprouting. *New Phytol.* 194 158–167. 10.1111/j.1469-8137.2011.04040.x 22300545

[B21] Ingversen J. Køie B. Doll H. (1973). Induced seed protein mutant of barley. *Cell. Mol. Life Sci.* 29 1151–1152. 10.1007/bf01946777

[B22] International Barley Genome Sequencing Consortium Mayer K. F. Waugh R. Brown J. W. Schulman A. Langridge P. (2012). A physical, genetic and functional sequence assembly of the barley genome. *Nature* 491 711–716. 10.1038/nature11543 23075845

[B23] Juhász A. Belova T. Florides C. G. Maulis C. Fischer I. Gell G. (2018). Genome mapping of seed-borne allergens and immunoresponsive proteins in wheat. *Sci. Adv.* 4:eaar8602. 10.1126/sciadv.aar8602 30128352 PMC6097586

[B24] Karlsson K. (1977). Linkage studies in a gene for high lysine content in Riso barley mutant 1508. *Barley Genet. Newslett.* 7 40–43.

[B25] Khakimov B. Rasmussen M. A. Kannangara R. M. Jespersen B. M. Munck L. Engelsen S. B. (2017). From metabolome to phenotype: GC-MS metabolomics of developing mutant barley seeds reveals effects of growth, temperature and genotype. *Sci. Rep.* 7:8195. 10.1038/s41598-017-08129-0 PMC5557882 28811511

[B26] Khan A. Mathelier A. (2017). Intervene: a tool for intersection and visualization of multiple gene or genomic region sets. *BMC Bioinformatics* 18:287. 10.1186/s12859-017-1708-7 28569135 PMC5452382

[B27] Kirk R. E. (1969). *Experimental Design: Procedures for the Behavioral Sciences. (Second Printing.).* Pacific Grove, CA: Brooks/Cole Publishing Company.

[B28] Kreis M. Shewry P. Forde B. Rahman S. Miflin B. (1983). Molecular analysis of a mutation conferring the high-lysine phenotype on the grain of barley (*Hordeum vulgare*). *Cell* 34 161–167. 10.1016/0092-8674(83)90146-0 6192931

[B29] Lange M. Vincze E. Wieser H. Schjoerring J. K. Holm P. B. (2007). Suppression of C-hordein synthesis in barley by antisense constructs results in a more balanced amino acid composition. *J. Agric. Food Chem.* 55 6074–6081. 10.1021/jf0709505 17580876

[B30] Liu Q. Guo Q. Akbar S. Zhi Y. El Tahchy A. Mitchell M. (2017). Genetic enhancement of oil content in potato tuber (*Solanum tuberosum* L.) through an integrated metabolic engineering strategy. *Plant Biotechnol. J.* 15 56–67. 10.1111/pbi.12590 27307093 PMC5253471

[B31] Marcotuli I. Houston K. Schwerdt J. G. Waugh R. Fincher G. B. Burton R. A. (2016). Genetic diversity and genome wide association study of β-glucan content in tetraploid wheat grains. *PLoS One* 11:e0152590. 10.1371/journal.pone.0152590 27045166 PMC4821454

[B32] Marzábal P. Gas E. Fontanet P. Vicente-Carbajosa J. Torrent M. Ludevid M. D. (2008). The maize Dof protein PBF activates transcription of γ-zein during maize seed development. *Plant Mol. Biol.* 67 441–454. 10.1007/s11103-008-9325-5 18379885

[B33] McCleary B. V. Draga A. (2016). Measurement of β-glucan in mushrooms and mycelial products. *J. AOAC Int.* 99 364–373. 10.5740/jaoacint.15-0289 26957216

[B34] McCleary B. V. Gibson T. S. Mugford D. C. (1997). Measurement of total starch in cereal products by amyloglucosidase-α-amylase method: collaborative study. *J. AOAC Int.* 80 571–579. 10.1093/jaoac/80.3.571

[B35] Mena M. Cejudo F. J. Isabel-Lamoneda I. Carbonero P. (2002). A role for the DOF transcription factor BPBF in the regulation of gibberellin-responsive genes in barley aleurone. *Plant Physiol.* 130 111–119. 10.1104/pp.005561 12226491 PMC166544

[B36] Mertz E. T. Bates L. S. Nelson O. E. (1964). Mutant gene that changes protein composition and increases lysine content of maize endosperm. *Science* 145 279–280. 10.1126/science.145.3629.279 14171571

[B37] Moehs C. P. Austill W. J. Holm A. Large T. A. Loeffler D. Mullenberg J. (2019). Development of reduced gluten wheat enabled by determination of the genetic basis of the lys3a low hordein barley mutant. *Plant Physiol.* 179 1692–1703. 10.1104/pp.18.00771 30696748 PMC6446766

[B38] Molberg Ø Solheim flÆte N. Jensen T. Lundin K. E. Arentz-Hansen H. Anderson O. D. (2003). Intestinal T-cell responses to high-molecular-weight glutenins in celiac disease. *Gastroenterology* 125 337–344. 10.1016/s0016-5085(03)00890-4 12891534

[B39] Munck L. Karlsson K. Hagberg A. Eggum B. O. (1970). Gene for improved nutritional value in barley seed protein. *Science* 168 985–987. 10.1126/science.168.3934.985 4909621

[B40] Nelson O. E. Mertz E. T. Bates L. S. (1965). Second mutant gene affecting the amino acid pattern of maize endosperm proteins. *Science* 150 1469–1470. 10.1126/science.150.3702.1469 17782299

[B41] Orman-Ligeza B. Borrill P. Chia T. Chirico M. Doležel J. Drea S. (2020). LYS3 encodes a prolamin-box-binding transcription factor that controls embryo growth in barley and wheat. *J. Cereal Sci.* 93:102965. 10.1016/j.jcs.2020.102965 32508376 PMC7263734

[B42] Patron N. J. Greber B. Fahy B. F. Laurie D. A. Parker M. L. Denyer K. (2004). The lys5 mutations of barley reveal the nature and importance of plastidial ADP-Glc transporters for starch synthesis in cereal endosperm. *Plant Physiol.* 135 2088–2097. 10.1104/pp.104.045203 15299120 PMC520780

[B64] R Core Team (2013). *R: A Language and Environment for Statistical Computing*. Vienna: R Foundation for Statistical Computing. Available online at: http://www.R-project.org/

[B43] Radchuk V. V. Borisjuk L. Sreenivasulu N. Merx K. Mock H.-P. Rolletschek H. (2009). Spatiotemporal profiling of starch biosynthesis and degradation in the developing barley grain. *Plant Physiol.* 150 190–204. 10.1104/pp.108.133520 19321714 PMC2675734

[B44] Ramírez-González R. Borrill P. Lang D. Harrington S. Brinton J. Venturini L. (2018). The transcriptional landscape of polyploid wheat. *Science* 361:eaar6089. 10.1126/science.aar6089 30115782

[B45] Rudi H. Uhlen A. K. Harstad O. M. Munck L. (2006). Genetic variability in cereal carbohydrate compositions and potentials for improving nutritional value. *Anim. Feed Sci. Technol.* 130 55–65. 10.1016/j.anifeedsci.2006.01.017

[B46] Schindelin J. Arganda-Carreras I. Frise E. Kaynig V. Longair M. Pietzsch T. (2012). Fiji: an open-source platform for biological-image analysis. *Nat. Methods* 9 676–682. 10.1038/nmeth.2019 22743772 PMC3855844

[B47] Shannon P. Markiel A. Ozier O. Baliga N. S. Wang J. T. Ramage D. (2003). Cytoscape: a software environment for integrated models of biomolecular interaction networks. *Genome Res.* 13 2498–2504. 10.1101/gr.1239303 14597658 PMC403769

[B48] Shewry P. Bunce N. Kreis M. Forde B. (1985). Polymorphism at the Hor 1 locus of barley (*Hordeum vulgare* L.). *Biochem. Genet.* 23 391–404. 10.1007/bf00499082 2994625

[B49] Shewry P. Faulks A. J. Miflin B. (1980). Effect of high-lysine mutations on the protein fractions of barley grain. *Biochem. Genet.* 18 133–151. 10.1007/bf00504365 6770843

[B50] Shewry P. Hm P. Mm L. Gj M. (1979). Protein metabolism in developing endosperms of high-lysine and normal barley. *Cereal Chem* 56 110–117.

[B51] Shewry P. R. (1993). *Barley: Chemistry and Technology*, eds Macgregor A. W. Bhatty R. S. (St Paul, MN: American Association of Cereal Chemists), 131–197.

[B52] Shewry P. R. Napier J. A. Tatham A. S. (1995). Seed storage proteins: structures and biosynthesis. *Plant Cell* 7:945. 10.2307/3870049 PMC160892 7640527

[B53] Sørensen M. (1992). Methylation of B-hordein genes in barley endosperm is inversely correlated with gene activity and affected by the regulatory gene Lys3. *Proc. Natl. Acad. Sci. U.S.A.* 89 4119–4123. 10.1073/pnas.89.9.4119 1570338 PMC525644

[B54] Tallberg A. (1977). The amino-acid composition in endosperm and embryo of a barley variety and its high lysine mutant. *Hereditas* 87 43–46. 10.1111/j.1601-5223.1977.tb01243.x

[B55] Tallberg A. (1981). Protein and lysine content in high-lysine double-recessives of barley II. Combinations between mutant 7 and a Hiproly back-cross. *Hereditas* 94 261–268. 10.1111/j.1601-5223.1981.tb01763.x

[B56] Tan Q. K.-G. Irish V. F. (2006). The Arabidopsis zinc finger-homeodomain genes encode proteins with unique biochemical properties that are coordinately expressed during floral development. *Plant Physiol.* 140 1095–1108. 10.1104/pp.105.070565 16428600 PMC1400567

[B57] Tanner G. J. Blundell M. J. Colgrave M. L. Howitt C. A. (2015). Creation of the first ultra-low gluten barley (*Hordeum vulgare* L.) for coeliac and gluten-intolerant populations. *Plant Biotechnol. J.* 14 1139–1150. 10.1111/pbi.12482 26427614 PMC5054857

[B58] Vanhercke T. El Tahchy A. Liu Q. Zhou X. R. Shrestha P. Divi U. K. (2014). Metabolic engineering of biomass for high energy density: oilseed-like triacylglycerol yields from plant leaves. *Plant Biotechnol. J.* 12 231–239. 10.1111/pbi.12131 24151938 PMC4285938

[B59] Venable J. D. Dong M.-Q. Wohlschlegel J. Dillin A. Yates J. R. (2004). Automated approach for quantitative analysis of complex peptide mixtures from tandem mass spectra. *Nat. Methods* 1:39. 10.1038/nmeth705 15782151

[B60] Xia J. Psychogios N. Young N. Wishart D. S. (2009). MetaboAnalyst: a web server for metabolomic data analysis and interpretation. *Nucleic Acids Res.* 37 W652–W660. 19429898 10.1093/nar/gkp356 PMC2703878

[B61] Yiming Y. Yidan O. Wen Y. (2018). shinyCircos: an R/Shiny application for interactive creation of Circos plot. *Bioinformatics* 34, 1229–1231. 10.1093/bioinformatics/btx763 29186362

[B62] Zhang Y. Cao X. Juhasz A. Islam S. Qi P. She M. (2018a). Wheat avenin-like protein and its significant Fusarium Head Blight resistant functions. *bioRxiv* [Preprint]. bioRxiv: 406694,

[B63] Zhang Y. Hu X. Islam S. She M. Peng Y. Yu Z. (2018b). New insights into the evolution of wheat avenin-like proteins in wild emmer wheat (*Triticum dicoccoides*). *Proc. Natl. Acad. Sci. U.S.A.* 115 13312–13317. 10.1073/pnas.1812855115 30530679 PMC6310801

