# Supplementary material for: Proteome and Nutritional Shifts Observed in Hordein Double-Mutant Barley Lines
Source: Front Plant Sci. 2021 Sep 9;12:718504. doi: 10.3389/fpls.2021.718504 (PMC8458801; doi:10.3389/fpls.2021.718504)
Supplement: Supplementary Figure 1 — Abundance profile of the 100 differentially abundant proteins across the WT, B-, C-, and BC-mutant lines. Heatmap showing the relative abundance levels (Z-scored) of significant altered proteins across the lines. Colour bars represent the abundance of proteins associated with GO functions such as fatty acid synthesis, nutrient reservoir and inhibitory activity. The bar charts (last three column) shows the log2 abundance of differentially abundant proteins in the WT and B-, C-, and BC-mutant lines. [file Data_Sheet_2.docx]

Supplementary Material

**Table S1: Hordein content of WT and hordein-single and double-mutant lines.**

| **Sample** | **µg/mL** | **Dilution x20** | **Protein (mg/mL)** |
| --- | --- | --- | --- |
| WT | 204 | 4943 | 4.09 |
| B-mutant | 104 | 2080 | 2.08 |
| C-mutant | 68 | 1379 | 1.37 |
| D-mutant | 198 | 3964 | 3.96 |
| BC-mutant | 187 | 3749 | 3.74 |
| BD-mutant | 135 | 2713 | 2.71 |
| CD-mutant | 124 | 2481 | 2.48 |


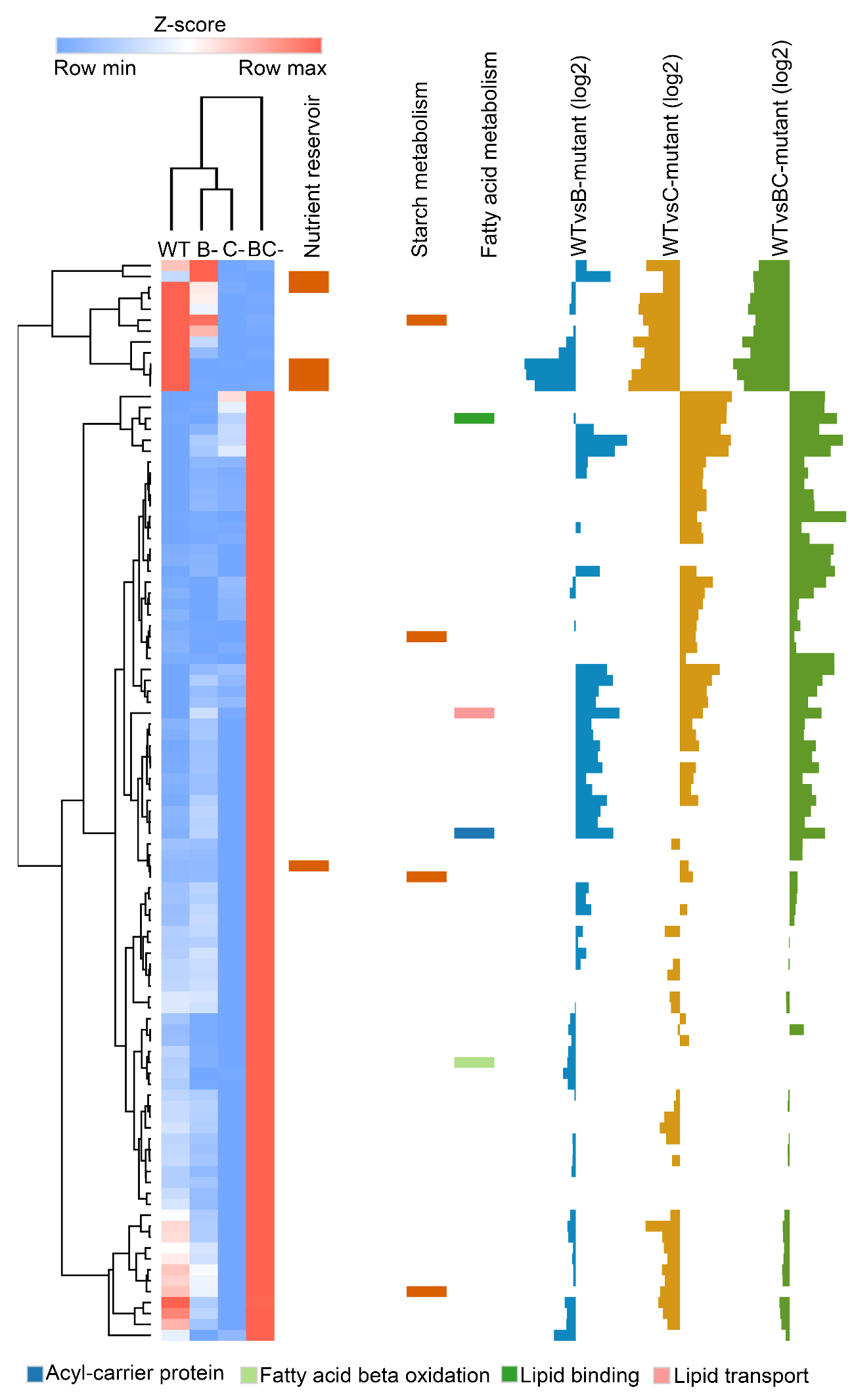


**Figure S1: Abundance profile of the 100 differentially expressed proteins across the WT, B-, C- and BC-mutant lines.** Heatmap showing the relative abundance levels (Z-scored) of significant proteins across the lines. Colour bars represent the abundance of proteins associated with GO functions such as fatty acid synthesis, nutrient reservoir and inhibitory activity. The bar charts (last three column) shows the log2 abundance of differentially abundant proteins in the WT and B-, C- and BC-mutant lines.


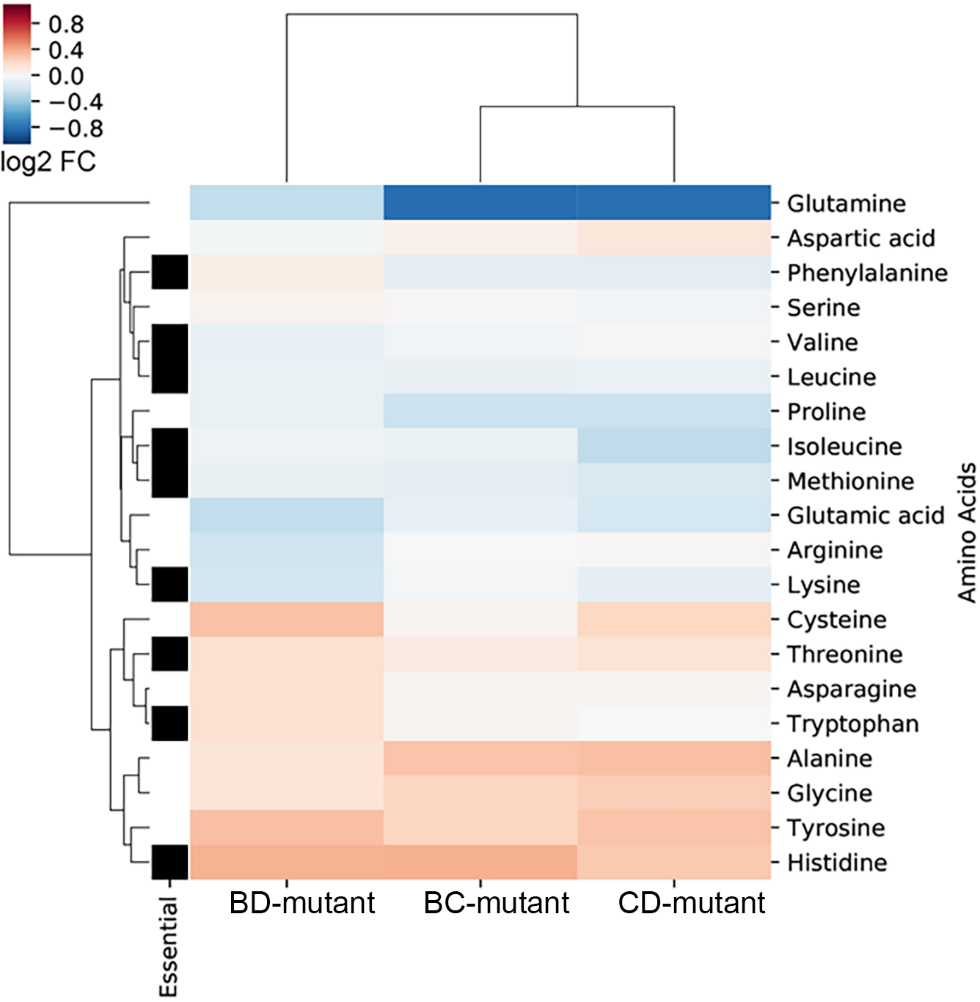


**Figure S2. Protein bound amino acid composition changes between double-mutant and WT lines.** Amino acid composition was determined for proteins significantly perturbed by hordein deletion and expressed as a fold change for the composition of proteins that are more abundant in mutant lines divided by the composition of proteins that are more abundant in the WT. These fold change values were log2 transformed for symmetry in the heat map where darker red indicates higher abundance in the mutant line and darker blue indicates higher abundance in the WT. Essential amino acids are indicated by black row side colouring.


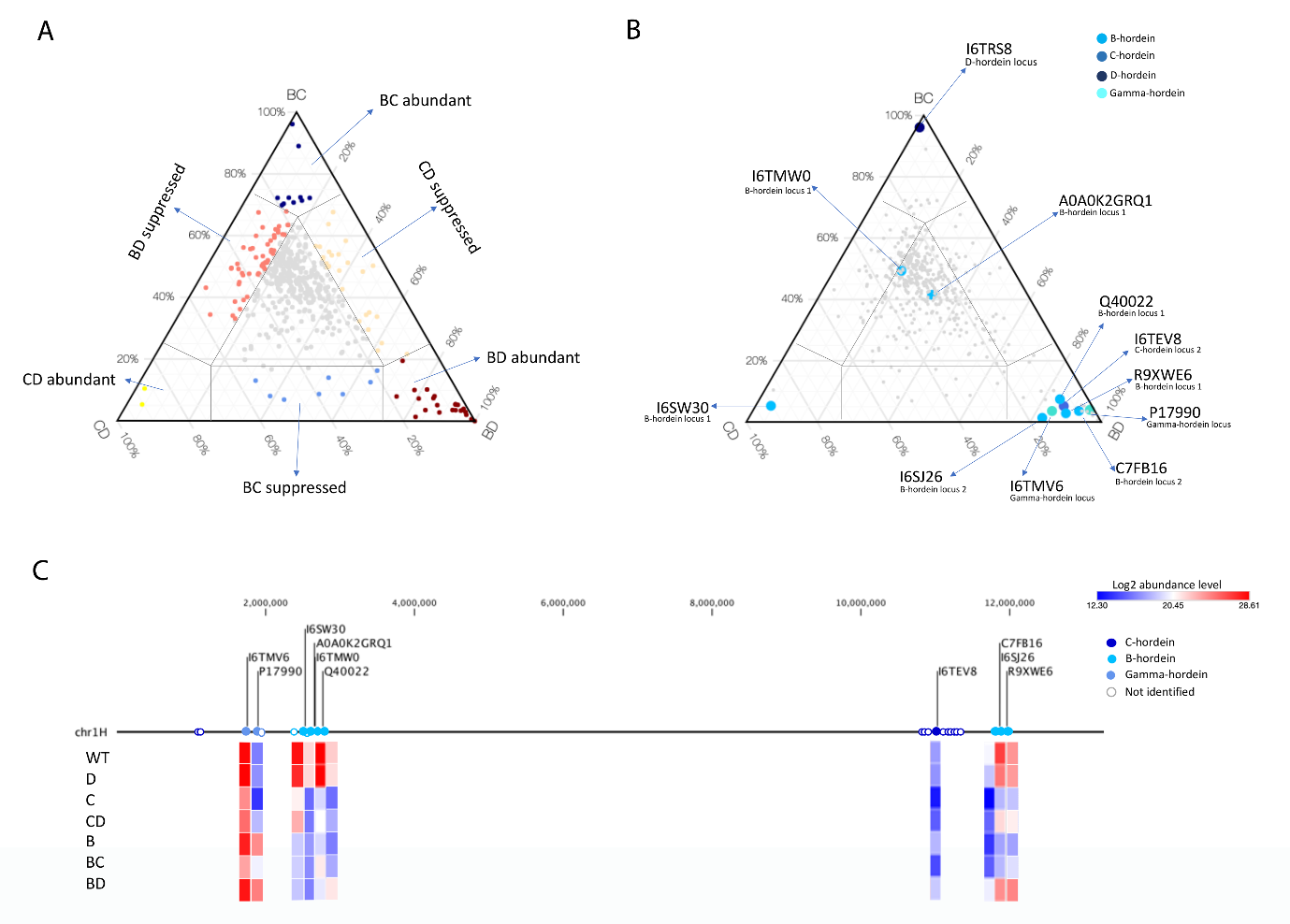


**Figure S3: (A)** Ternary plot representing relative abundance comparison of the three double-mutants. Abundance bias categories highlighting DN dominant, DN suppressed and balanced abundance are presented. (**B**) Ternary plot highlighting relative abundance values of the analysed hordeins. Mapped hordein locus positions are also labelled. (**C**) Genomic location of barley hordeins indicate the presence of multiple B- and C- hordein loci on the short arm of chromosome 1H (**Fig 6**). The first C-hordein locus is located at 1.45 Mb and is composed of at least two C-hordein genes. This locus is followed by the gamma-hordein locus at 1.75 Mb and is composed of at least of 3 gamma-hordein genes. The B hordein locus 1 spans between 2.52 and 3.25 Mb. The second C-hordein locus covers a 200 kb region between 11 and 11.2 Mb and this followed by another set of B-hordein genes at 11.95 Mb. In the double-mutants we have mapped two hordein sequences to the gamma hordein locus, six hordein sequences to the B-hordein locus 1, two sequences to the C-hordein locus 2 and two sequences to the B-hordein locus 2.


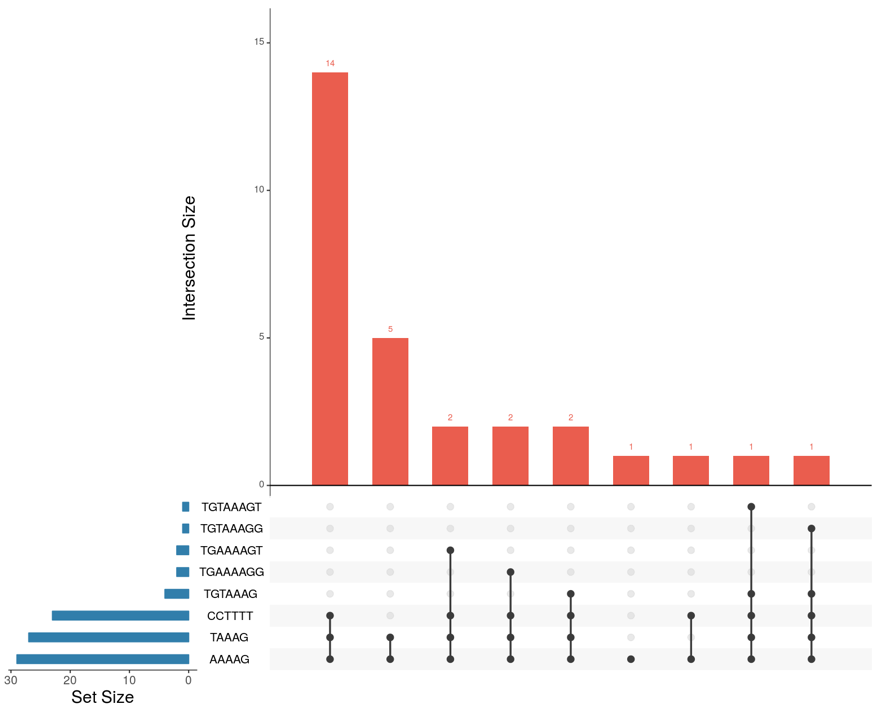


**Figure S4.** Enrichment of Prolamin box variants (TGTAAAGT, TGTAAAGG, TGAAAAGT, TGAAAAGG, TGTAAAG), DOF-core motif (A/TAAG) and Pyrimidine-box (CCTTTT) motifs.
