# Supplementary material for: Proteome and Nutritional Shifts Observed in Hordein Double-Mutant Barley Lines
Source: Front Plant Sci. 2021 Sep 9;12:718504. doi: 10.3389/fpls.2021.718504 (PMC8458801; doi:10.3389/fpls.2021.718504)
Supplement: Supplementary File 1 — Detailed materials and methods for embryo size measurement, barley β-glucan analysis, total starch content analysis, total fatty acid, and total TAG content analysis and circus plot. [file Data_Sheet_1.docx]

**File S1**

**Proteome and nutritional shifts observed in hordein double-mutant barley lines**

Utpal Bose^1,2‡^, Angéla Juhász^2‡^, Ronald Yu^3^, Mahya Bahmani^2^, Keren Byrne^1^, Malcolm Blundell^3^, James A. Broadbent^1^, Crispin A. Howitt^3^, Michelle L. Colgrave^1,2*^

^1^CSIRO Agriculture and Food, 306 Carmody Rd, St Lucia QLD 4067, Australia; ^2^Australian Research Council Centre of Excellence for Innovations in Peptide and Protein Science, School of Science, Edith Cowan University, Joondalup WA 6027, Australia, ^3^CSIRO Agriculture and Food, GPO Box 1700, Canberra ACT 2601, Australia

^‡^Both authors contributed equally

^*^Corresponding author: Michelle L. Colgrave, CSIRO Agriculture and Food, 306 Carmody Rd, St Lucia QLD 4067, Australia; Phone: +61 7 3214 2697; Fax: +61 7 3214 2900; Email: michelle.colgrave@csiro.au

**Materials and methods**

**Embryo size measurement**

Macro functions were used to apply filters and segment the whole barley area, which was then measured by the pixel measurement tool in Fiji. The embryo area was also manually selected from the unprocessed image and measured. Embryo size was represented by the proportion of embryo area with regards to the whole barley grain. Statistical analyses were conducted using R, Tukey’s HSD test.

**Barley β-glucan analysis**

Firstly, 200 µL of 50% ethanol was added to 20 mg of barley flour, followed by addition of 1 mL of sodium phosphate buffer (20 mM, pH 6.5) with incubation at 100°C for 3 min. After brief cooling, 1 mL of distilled water was added to the samples with incubation at 100°C for another 3 min, before allowing to cool in a 40°C water bath for 30 min. Secondly, 10 µL lichenase (1 U/µL) was added with incubation at 40°C for 1 hour. After the lichenase reaction, 3.8 mL distilled water was added to the sample, mixed well, and centrifuged at 2,000 x g for 10 min. Thirdly, three aliquots of 10 µL of sample supernatant was added to a 96-well plate (wherein the blank consisted of 10 µL of sodium phosphate buffer), followed by addition of 10 µL of β-glucosidase (2 U/mL). The reactions were incubated at 40°C for 15 min.

After the enzymatic digestion, the released glucose was quantified. Firstly, 150 µL of glucose oxidase/peroxidase (GOPOD) reagent was added to each 20 µL sample, the reaction was then incubated at 40°C for 20 min. Secondly, the absorbance was measured at 510 nm for each sample (EA) and reagent blank (EBLANK). The β-glucan content was measured using the following formula:

β-glucan (% weight/total weight) = ∆E × F x 600 × 1/1000 x 100/Wd × 162/180

= ∆E / ∆G / Wd × 135

where ∆E is the difference between EA and EBLANK, F is the absorbance of 2.5 µg of glucose, and Wd is the weight of sample analysed (in mg) multiplied by 0.86 (Wd x 0.86).

**Total starch content analysis**

Samples of 0.1 g barley flour were mixed with 80% ethanol, followed by the addition of 3 mL of thermostable α-amylase (1:30 dilution in 50 mM MOPS buffer, pH 7.0; K-CERA, Megazyme) with incubation at 100°C boiling water bath for 6 min. The samples were then cooled in a 50°C water bath, followed by additions of 4 mL of sodium acetate buffer (200 mM, pH 4.5) and 0.1 mL of amyloglucosidase (R-AMGR3, Megazyme) with incubation at 50°C for 30 min. After the reaction, the samples were diluted with distilled water up to 10 mL and centrifuged at 2,500 xg for 10 min. The released D-glucose was then oxidised to D-gluconate and hydrogen peroxide, in which the amount of hydrogen peroxide released was quantified by the production of the quinoneimine dye from the subsequent peroxidation reaction. After the centrifugation, duplicate aliquots of the supernatant were mixed with 3 mL GOPOD reagent with incubation at 50°C for 20 min. The absorbance at 510 nm of each sample, the blank and the 1 mg/mL D-glucose standard were determined.

The starch content was calculated according to the below equation.

Starch content=∆A*(100/S)*(FV/0.1)*(1/1000)*(100/W)*(162/180)

where ∆A represents the difference in absorbance at 510 nm between reaction and blank, S represents the absorbance of the standard of 100 µg of D-glucose, FV represents the final volume with the dilution of distilled water after the amylase and amyloglucosidase reactions, and W represents the weight of rice flour sample.

**Total fatty acid and total TAG content analysis**

Briefly, neutral lipid and free fatty acid were separated on thin layer chromatography (TLC) in a solvent matrix consisting of hexane : diethyl ether : acetic acid in 70:30:1 volume ratio while polar lipids were separated by TLC in a matrix of chloroform : methanol : acetic acid : distilled water in 90:15:10:3. The TLC- separated lipid was then harvested and extracted, followed by subsequent derivatisation to fatty acid methyl esters (FAME) and gas chromatography (GC) analyses.

**Circos plot**

To visualize the abundance changes of the mapped proteins across the double mutants a Circos plot analysis was performed. First, Pearson correlation values were calculated for each protein pairs using the mean protein abundance values. Pearson correlation values above 0.95 and below -0.95 were used both in protein-protein interaction network analysis and to visualize potential interactions in relation to their chromosomal location.

ShinyCircos, an R/Shiny application was used to construct chromosome based circos plot (Yu *et al.*, 2018). Log 2 adjusted protein abundance values are highlighted in the heatmap panel. Hordeins, avenin-like proteins and proteins with fatty acid degradation, fatty acid metabolism or starch metabolism function are labelled. Potential protein-protein interactions defined based on the Pearson correlation value were visualised.

**References**

Yu Y, Ouyang Y, & Yao W. 2018. shinyCircos: an R/Shiny application for interactive creation of Circos plot. *Bioinformatics* **34,** 1229-1231.
